# Implementing a Patient Portal for the Remote Follow-Up of Self-Isolating Patients With COVID-19 Infection Through Patient and Stakeholder Engagement (the Opal-COVID Study): Mixed Methods Pilot Study

**DOI:** 10.2196/48194

**Published:** 2024-12-04

**Authors:** Yuanchao Ma, David Lessard, Serge Vicente, Kim Engler, Adriana Rodriguez Cruz, Moustafa Laymouna, Opal-COVID Patient Expert Committee, Tarek Hijal, Lina Del Balso, Guillaume Thériault, Nathalie Paisible, Nadine Kronfli, Marie-Pascale Pomey, Hansi Peiris, Sapha Barkati, Marie-Josée Brouillette, Marina Klein, Joseph Cox, Alexandra de Pokomandy, Jamil Asselah, Susan J Bartlett, Bertrand Lebouché

**Affiliations:** 1 Center of Outcomes Research and Evaluation Research Institute of the McGill University Health Centre Montreal, QC Canada; 2 Infectious Diseases and Immunity in Global Health Program Research Institute of McGill University Health Centre Montreal, QC Canada; 3 Chronic Viral Illness Service McGill University Health Centre Montreal, QC Canada; 4 Department of Biomedical Engineering Polytechnique Montréal Montreal, QC Canada; 5 Department of Mathematics and Statistics University of Montreal Montreal, QC Canada; 6 Department of Family Medicine Faculty of Medicine and Health Sciences McGill University Montreal, QC Canada; 7 See Acknowledgments; 8 Department of Radiation Oncology McGill University Health Centre Montreal, QC Canada; 9 Department of Medicine Faculty of Medicine and Health Sciences McGill University Montreal, QC Canada; 10 Research Centre of the University of Montreal Hospital Centre Montreal, QC Canada; 11 Centre of Excellence on Partnership with Patients and the Public Montreal, QC Canada; 12 Department of Health Policy, Management and Evaluation School of Public Health University of Montreal Montreal, QC Canada; 13 Department of Psychiatry Faculty of Medicine and Health Sciences McGill University Montreal, QC Canada; 14 Department of Epidemiology, Biostatistics and Occupational Health Faculty of Medicine and Health Sciences McGill University Montreal, QC Canada; 15 Department of Medicine Division of Medical Oncology McGill University Health Centre Montreal, QC Canada

**Keywords:** SARS-CoV-2, coronavirus, infectious disease, implementation science, Canada, patient portal, telehealth, telemedicine, app, health information technology, remote monitoring, mobile phone

## Abstract

**Background:**

The COVID-19 pandemic was an unprecedent challenge to public health systems, with 95% of cases in Quebec sent home for self-isolation. To ensure continuous care, we implemented an intervention supported by a patient portal (Opal) to remotely monitor at-home patients with COVID-19 via daily self-reports of symptoms, vital signs, and mental health that were reviewed by health care professionals.

**Objective:**

We describe the intervention’s implementation, focusing on the (1) process; (2) outcomes, including feasibility, fidelity, acceptability, usability, and perceived response burden; and (3) barriers and facilitators encountered by stakeholders.

**Methods:**

The implementation followed a co-design approach operationalized through patient and stakeholder engagement. The intervention included a 14-day follow-up for each patient. In the mixed methods study at the McGill University Health Centre in Montreal, Quebec, participants completed questionnaires on implementation outcomes on days 1, 7, and 14. All scores were examined against predefined success thresholds. Linear mixed models and generalized estimating equations were used to assess changes in scores over time and whether they differed by sex, age, and race. Semistructured interviews were conducted with expert patients, health care professionals, and coordinators for the qualitative analysis and submitted to thematic analysis guided by the Consolidated Framework for Implementation Research.

**Results:**

In total, 51 participants were enrolled between December 2020 and March 2021; 49 (96%) were included in the quantitative analysis. Observed recruitment and retention rates (51/52, 98% and 49/51, 96%) met the 75% feasibility success threshold. Over 80% of the participants found it “quite easy/very easy” to complete the daily self-report, with a completion rate (fidelity) of >75% and a nonsignificant decreasing trend over time (from 100%, 49/49 to 82%, 40/49; *P*=.21). Mean acceptability and usability scores at all time points exceeded the threshold of 4 out of 5. Acceptability scores increased significantly between at least 2 time points (days 1, 7, and 14: mean 4.06, SD 0.57; mean 4.26, SD 0.59; and mean 4.25, SD 0.57; *P*=.04)*.* Participants aged >50 years reported significantly lower mean ease of use (usability) scores than younger participants (days 1, 7, and 14: mean 4.29, SD 0.91 vs mean 4.67, SD 0.45; mean 4.13, SD 0.89 vs mean 4.77, SD 0.35; and mean 4.24, SD 0.71 vs mean 4.72, SD 0.71; *P*=.004). In total, 28 stakeholders were interviewed between June and September 2021. Facilitators included a structured implementation process, a focus on stakeholders’ recommendations, the adjustability of the intervention, and the team’s emphasis on safety. However, Opal’s thorough privacy protection measures and limited acute follow-up capacities were identified as barriers, along with implementation delays due to data security–related institutional barriers.

**Conclusions:**

The intervention attained targets across all studied implementation outcomes. Qualitative findings highlighted the importance of stakeholder engagement. Telehealth tools have potential for the remote follow-up of acute health conditions.

**International Registered Report Identifier (IRRID):**

RR2-10.2196/35760

## Introduction

### Background

COVID-19 is a major public health concern. At the beginning of the pandemic in 2020, the large number of patients attending clinics for screening and treatment posed unprecedented challenges for hospital management [1,2]. To allow hospitals to focus on patients considered vulnerable and seriously ill, 95% of those with COVID-19 infection in Quebec were sent home for self-isolation and self-care. During this period, the clinical features of people with COVID-19 infection were well known: most presented mild or no symptoms during the first week, but some deteriorated rapidly within hours to days in the second week [3,4]. When a patient’s condition worsened, delays in identification and treatment could lead to poor patient outcomes, including death. Self-isolation was thus a source of anxiety and distress, especially for people at risk of deterioration, such as older adults and those living alone or with chronic conditions [5,6].

To ensure continuous care and to address some of the psychological implications of self-isolation [7], it became crucial to help self-isolating patients with COVID-19 infection monitor their health condition and maintain contact with health care professionals. Telehealth, which uses telecommunication technologies to deliver care and health services, can address some of these challenges, empower its users, and efficiently support self-management of care by enabling patients to collect and remotely share health information with health care professionals [8,9].

For this purpose, Opal was a promising telehealth tool. Currently used by >5000 patients, it is an award-winning patient portal first implemented in the radiation-oncology department of the McGill University Health Centre (MUHC) in 2018 [10]. A patient portal is a connected platform (eg, website, software, and app) that gives patients access to a portion of their electronic medical records, such as their appointment calendar, laboratory results, and health care provider’s clinical notes. Co-designed by expert patients [11], IT developers, and health care professionals, Opal also provides, through a smartphone app available in English and French, educational materials and patient-reported outcome measures (PROMs) for completion [12]. Physicians use Opal to remotely administer these through a desktop dashboard.

### Objectives

When COVID-19 was declared a global pandemic, our team was working on implementing Opal in HIV clinical care. We redirected these efforts to support self-isolating patients with COVID-19 infection, while helping them avoid face-to-face interactions with health care professionals [13]. This paper aims to describe the intervention’s implementation, focusing on the (1) process; (2) outcomes, including feasibility, fidelity, acceptability, usability, and perceived response burden; and (3) barriers and facilitators from the perspective of stakeholders. The assessed clinical outcomes will be presented in another manuscript.

## Methods

### Implementation Strategy

#### Overview

This study followed the principles of co-design, which refers to creative cooperative processes involving diverse experts and potential end users during the planning and development stages of products, projects, or interventions [14,15]. Collaboration in defining expectations and solutions can optimize the implementation and outcomes of a telehealth-based intervention [13].

We operationalized co-design through patient and stakeholder engagement (PSE), that is, the meaningful involvement of stakeholders affected by a given health condition and its associated care, such as patients, health care professionals, and researchers, in potentially all steps of a given project. PSE seeks the coconstruction of knowledge [16-19] by emphasizing deliberation in health-related decision-making [20,21], patient autonomy [22], sensitive listening and accountability, and partnerships [23]. PSE has been critical in the context of the COVID-19 pandemic to ensure that research effectively and rapidly translated into social and medical benefits [24]. Our researcher-driven PSE framework [18,25,26] represents activities ranging from (1) information (informing patients and stakeholders) and (2) consultation (obtaining and accounting for patients’ and stakeholders’ perspectives) to (3) collaboration (partnering with patients and stakeholders in decision-making) [27-30].

**Figure 1:**
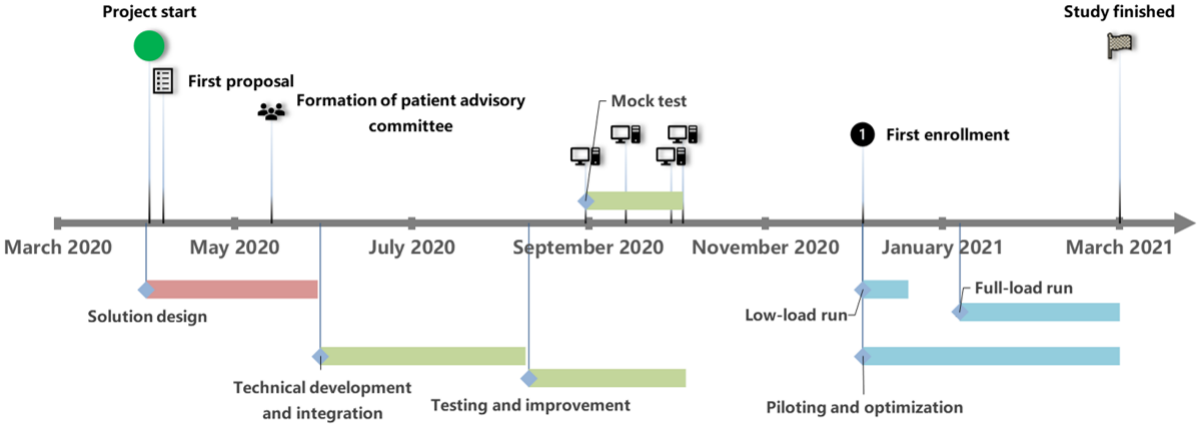
Opal-COVID solution configuration and implementation timeline.

In this project, PSE included three phases (Figure 1): (1) solution design; (2) technical integration and testing; and (3) a pilot study phase, including a low-load and a full-load run, which are defined in the Study Design and Recruitment subsection.

#### Solution Design (April-May 2020)

The initial research team included 6 members: BL (MD, PhD, principal investigator [PI], and clinician-scientist), a COVID-19 frontline physician; KE (PhD in public health) and Kedar KV Mate (PhD in neurophysiotherapy), both experts in PROM development; DL (PhD in anthropology), an expert in PSE; ARC (PhD in immunology), the research coordinator; and YM (MSc in engineering), the technical coordinator.

The research team recognized the possibility of using Opal to follow patients with COVID-19 infection and secured funding from the McGill Interdisciplinary Initiative in Infection and Immunity Emergency COVID-19 Research Funding (ECRF-R2-44) on April 20, 2020. BL and YM confirmed their intention to implement Opal for the clinical follow-up of self-isolating patients with COVID-19 infection with its IT developers led by TH and John Kildea. BL, YM, KE, and Kedar KV Mate then conducted a week-long first set of meetings to broadly identify the target population for the intervention, the proposed follow-up, and its overall mechanisms.

Subsequently, BL, DL, and YM organized a series of meetings with the Opal–COVID-19 expert patient committee consisting of 3 patients who had recovered from COVID-19 infection, as well as nurses (LDB and GT) and physicians, including infectious disease and public health specialists (NK, SB, JC, and MK) and a psychiatrist (MJB). During these meetings, stakeholders made recommendations for the intervention, the selection of PROMs, and other data collection instruments.

Researchers and stakeholders consensually decided that patients would use Opal to self-report symptoms, vital signs, and mental health daily using validated instruments (Multimedia Appendix 1). Responses to certain questions could trigger symptom management counseling that provided guidance to patients; for example, if a patient lost their sense of smell/taste, they would be advised to avoid using inhaled or oral corticosteroids for treatment and to contact the nurse for more information. Nurses would review the results remotely and send appropriate feedback based on their observations. Nurses could, for example, confirm that a patient’s health status was stable or offer a teleconsultation with a physician. The intervention lasted a minimum of 14 days and could be extended if the patient’s condition required further follow-up (eg, persistent symptoms). Medical devices (ie, pulse oximeters and thermometers) were sent to patients who did not have them, and educational materials (eg, government guidelines and instructional videos for medical devices) were prepared and integrated into Opal.

In the meantime, KE and Kedar KV Mate collaborated to prepare a pilot study protocol for this project.

#### Technical Integration and Testing (June-November 2020)

YM initiated ongoing exchanges with Opal developers through email and videoconferencing to discuss the project requirements and the technical aspects of configuring Opal for managing patients with COVID-19 infection. They followed the Agile framework [31] for project management and software development, which was used during the initial development of Opal for managing patients with cancer [12]. The Agile framework consists of an iterative approach interspersing episodes of technical work on specific aspects of software with testing and debriefing with stakeholders and testers for feedback.

First, the IT developers adapted the clinical dashboard to make it easier for nurses to access and track patients’ self-reports. They also created and integrated electronic versions of the selected PROMs and data collection tools into Opal, along with educational materials. They configured standardized feedback messages to patients, based on their daily self-report results, and relevant appointment information.

Four prototype tests of functionality and usability were then conducted over a 2-month period. Before each test, YM provided a videoconference training session to health care professionals and expert patient committee members, introducing them to the latest improvements in Opal. The expert patients tested Opal on their personal device for 4 days and provided feedback (eg, reporting bugs and suggesting improvements), while health care professionals made recommendations concerning the dashboard. On the basis of stakeholder feedback, developers adjusted Opal after each test; for example, we integrated a color code (red and green) to encourage patient completion of essential aspects of the self-report. In addition, YM developed guidelines for health care professionals participating in the pilot study with the input received during testing.

#### Piloting and Optimization (December 2020-March 2021)

This phase encompasses the pilot study conducted at the MUHC (Glen site) in Montreal, Quebec. This study was registered in ClinicalTrials.gov (NCT04978233). This study and its results are described in the sections that follow. Three frontline nurses (GT, NP, and LDB), a technical coordinator (YM), and a research coordinator (ARC) ensured that the pilot testing was conducted effectively, and an on-call physician was available for consultation if necessary. Each nurse monitored and followed up to 6 patient participants simultaneously.

Throughout the pilot study, feedback from users, including patient participants and health care professionals, was recorded in a coordinator logbook. Accordingly, the intervention and implementation were optimized, promoting adaptability. The adjustments included (1) modifying the self-report by changing some questions from mandatory to optional and adding a “none of the above” option to certain multiple-choice questions, (2) implementing additional daily self-reports for participants with symptoms that required further observation, (3) proactively contacting participants who did not respond for 3 consecutive days, and (4) applying hotfixes for bugs encountered during the use of the registration system and clinical monitoring dashboard (eg, nonfunctional registration code system and incorrect last check time for the questionnaire).

### Study Design and Recruitment

This pilot study used mixed quantitative and qualitative methods to report implementation strategies and evaluate outcomes. The previously published protocol [32] contains additional methodological details on the study and its intervention. We reported our findings by following the guidance provided by the CONSORT (Consolidated Standards of Reporting Trials) statement for pilot and feasibility studies (Multimedia Appendix 2) [33,34] and the StaRI (Standards for Reporting Implementation Studies; Multimedia Appendix 3) [35].

Participants were selected using convenience sampling [36]. Participant inclusion criteria were as follows: aged ≥18 years, fluent in French or English, testing positive for COVID-19 infection at the MUHC and being instructed to self-isolate, enrollment in Quebec’s provincial health insurance plan, comfortable with using health-related apps via a smart device (eg, a smartphone) or having someone close by who is, and possessing an internet connection. Exclusion criteria were being hospitalized, concurrent enrollment in another COVID-19 clinical trial, or having a cognitive impairment that prevented participation.

When delivering a positive SARS-CoV-2 infection test result, the MUHC test center staff briefly explained the study to the patient and asked whether they were interested in participating. The study coordinator then contacted interested individuals either on the same day or the following day to schedule a videoconference appointment to obtain consent. After consent, the technical coordinator helped participants register on the Opal app and offered training. Recruitment began with a “low-load” run of 5 participants recruited from early to mid-December 2020. Recruitment for the “full-load” run began in January 2021 to include the remainder of the quantitative study sample.

One month after all patient follow-up ended with Opal, we used purposive expert sampling [25] to propose to expert patients, health care professionals, and the coordinators of the study (ie, IT developers and study staff) involved in any of the 3 phases of the implementation to participate in a qualitative interview. A research coordinator sent an email to these stakeholders with an invitation to schedule a qualitative interview. All agreed to be interviewed.

### Quantitative Data

#### Data Collection

The pilot study participants completed a 1-time sociodemographic questionnaire on day 1 and a research questionnaire each week (ie, on days 1, 7, and 14 of the intervention) on implementation outcomes, namely acceptability, usability, and perceived response burden. To supplement the assessment of usability, participants were asked in the daily self-report whether they had help to complete the self-report. Recruitment data and completion records of the daily self-reports were recorded in the coordinator logbook to assess feasibility and fidelity. Success thresholds, outlined in the following paragraphs, were set for each outcome.

To assess feasibility, that is, how successfully an intervention can be used in a given setting [37], we examined the recruitment rate (ie, the proportion of eligible contacts enrolled in the study), and the retention rate (ie, the proportion of participants who remained enrolled for the whole duration of the intervention), both with a target of at least 75% [38].

We evaluated fidelity, the degree to which the intervention was implemented as intended [37], by measuring the proportion of participants who filled out the daily self-reports (completion rate) every day for the duration of the intervention, also with a minimum target of 75%.

To evaluate acceptability, which reflects how agreeable, palatable, or satisfactory an intervention is perceived to be [37], we adapted the Acceptability of Intervention Measure (Multimedia Appendix 4) [39]. This scale consists of 6 items rated on a 5-point Likert scale, with scores averaged to produce a summary score ranging from 1 to 5. In accordance with the recommendations of the scale developers, a minimum average score of 4 was considered indicative of high acceptability.

To assess usability, the extent to which using a product can achieve specific goals [40], we used the Health Information Technology Usability Evaluation Scale (Multimedia Appendix 4) [41]. This scale is customizable and specifically designed to evaluate telehealth technology. We selected subscales concerned with perceived impact (3 items), usefulness (9 items), and ease of use (5 items). The items were rated on a 5-point scale of agreement and averaged to generate subscale scores ranging from 1 to 5. Consistent with the previous threshold, the target was a mean score of at least 4 on each subscale.

Finally, we evaluated perceived response burden, represented by the effort required to answer the daily self-reports on Opal, by adapting a single question with a 5-point response scale from an existing survey, with scores ranging from 1 to 5 (Multimedia Appendix 4) [42]. We considered ≥80% of the participants rating the perceived response burden as “quite easy” or “very easy” as a success.

#### Statistical Analysis

The sample’s sociodemographic characteristics were described using frequency counts and proportions. These factors are deemed important because patient portal use varies by sociodemographic factors [9,43,44]. Indeed, capturing sex, age, and racial group is essential in portal research to assess generalizability [45]. Hence, the implementation outcomes were summarized with descriptive statistics and stratified by the selected sociodemographic variables (sex, age, and racial group) at days 1, 7 and 14. Acceptability and usability, treated as continuous outcomes, were summarized using the minimum, the maximum, and mean (SD). Feasibility, fidelity, and perceived response burden, treated as ordinal outcomes, were summarized using frequency counts and proportions.

We used linear mixed models to evaluate whether mean acceptability and usability scores changed significantly over time. The dependent variable for each model was the implementation outcome considered, and the independent variable was time (days 1, 7, and 14). If, at each time point, the outcome’s mean score was greater than or equal to the predefined success threshold, we considered that the target was met. If not, we used a 1-tailed *t* test to test the null hypothesis of threshold nonattainment.

The evaluation of perceived response burden was similar to that of acceptability and usability but with parameters estimated using generalized estimating equations for ordinal data. To test the null hypothesis of threshold nonattainment when the observed proportion fell below the predefined success threshold, we used a 1-tailed *z* test.

To evaluate whether fidelity changed significantly over time, we used a generalized estimating equations model for binary data. The dependent variable was the completion rate, and the independent variable was time (days 1 to 14). To test the null hypothesis of threshold nonattainment when the observed completion rate fell below the predefined success threshold, we used a 1-tailed *z* test.

Finally, the analysis was repeated with the selected sociodemographic variables added separately as independent variables to determine whether there were significant differences in the associated implementation outcomes between the groups represented over time.

For all hypothesis tests, the significance level was set at 5%.

### Qualitative Data

#### Data Collection

To better understand stakeholder experiences of the implementation process, we conducted qualitative interviews with the stakeholders via videoconferencing, using either Zoom (Zoom Video Communications, Inc) or Skype (Microsoft Corp). Each interview lasted 30 to 45 minutes and was recorded. Trained and experienced researchers conducted them in French or English following a semistructured guide (Multimedia Appendix 5) on the following themes: experiences with COVID-19 or providing COVID-19–related care, role in the intervention’s implementation, perspective on the intervention, and recommendations for improvement. The interview guide included follow-up questions on each main theme.

#### Analysis

The interview recordings were transcribed verbatim and deidentified, after which they were reviewed by DL. DL and ML conducted an inductive-deductive thematic analysis [46] using NVivo 12 (Lumivero). They used the Consolidated Framework for Implementation Research (CFIR) [47], a commonly used implementation science framework, to identify influences on implementation. Using the CFIR’s 5 broad domains and 39 constructs, DL and ML deductively coded and categorized interview content on the stakeholders’ experiences of the intervention and its implementation. They used these categories to identify themes associated with CFIR domains. Illustrative quotes in French presented in this manuscript have been translated into English. To ensure reliability, the results were repeatedly discussed with coauthors.

### Ethics Approval

This study was approved by the MUHC Research Ethics Board (2021-6763).

## Results

### Quantitative Results

#### Sociodemographic Characteristics

**Figure 2:**
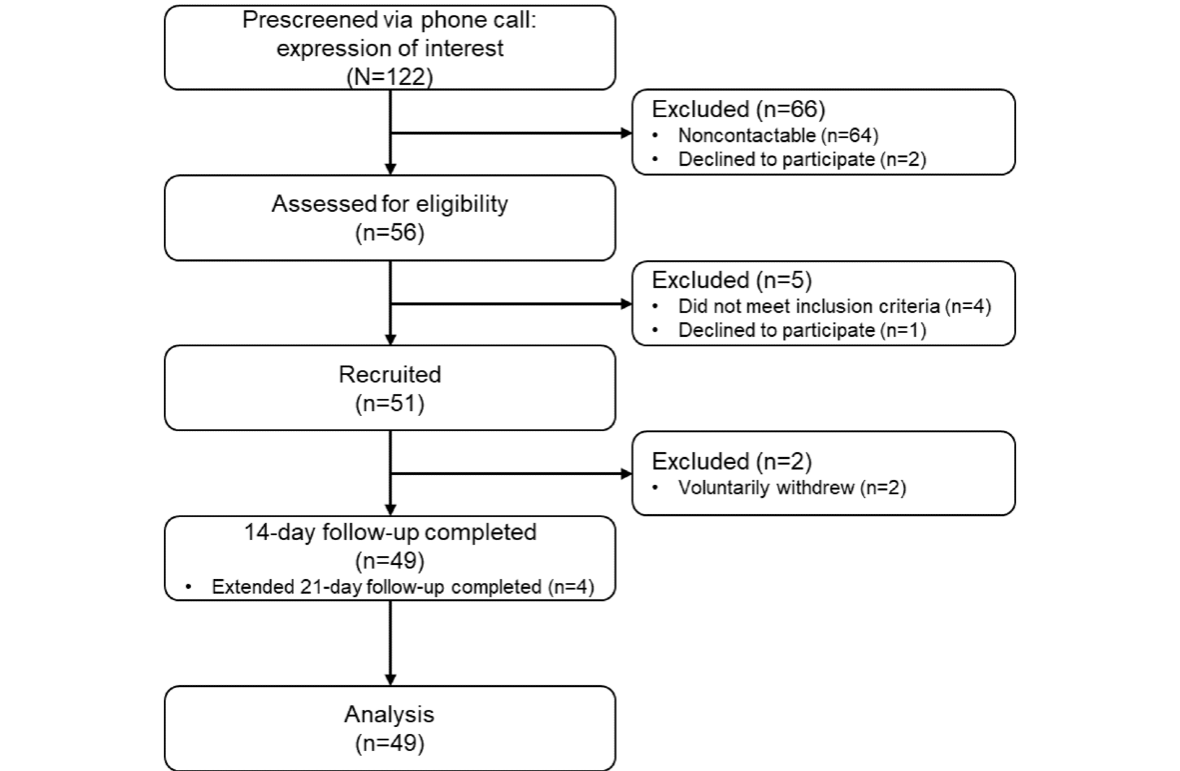
Opal-COVID study participant flowchart.

Figure 2 shows the flow of participants through the pilot study. From December 8, 2020, to February 23, 2021, a total of 51 patients were enrolled in the study. Of these 51 patients, 2 (4%) withdrew voluntarily before day 14, while 45 (88%) completed the 14-day follow-up, and 4 (8%) had their follow-up extended to 21 days because they were still symptomatic at day 14. All participants who completed at least 14 days of follow-up (49/51, 96%) were included in the analysis.

The sociodemographic characteristics of the study sample are described in Multimedia Appendix 6. As more than half of the participants (25/49, 51%) chose not to disclose their income, this variable was not reported. Table 1 displays the selected sociodemographic variables considered for further statistical analysis. As 3 (6%) of the 49 participants did not provide sociodemographic information, the sample size for analysis with these variables was 46.

**Table 1:** Descriptive statistics of the sociodemographic variables considered for statistical analysis (n=46).

Characteristics			Participants, n (%)
**Sex**			
	Female	23 (50)	
	Male	23 (50)	
**Age group (years)**			
	18-50	37 (80)	
	51-70	9 (20)	
**Racial group**			
	White	22 (48)	
	People of color	24 (52)	

#### Feasibility, Fidelity, and Usability

In terms of feasibility, the observed recruitment rate was 98% (51/52) and the retention rate was 96% (49/51), both of which were above the predetermined success threshold of 75%.

**Figure 3:**
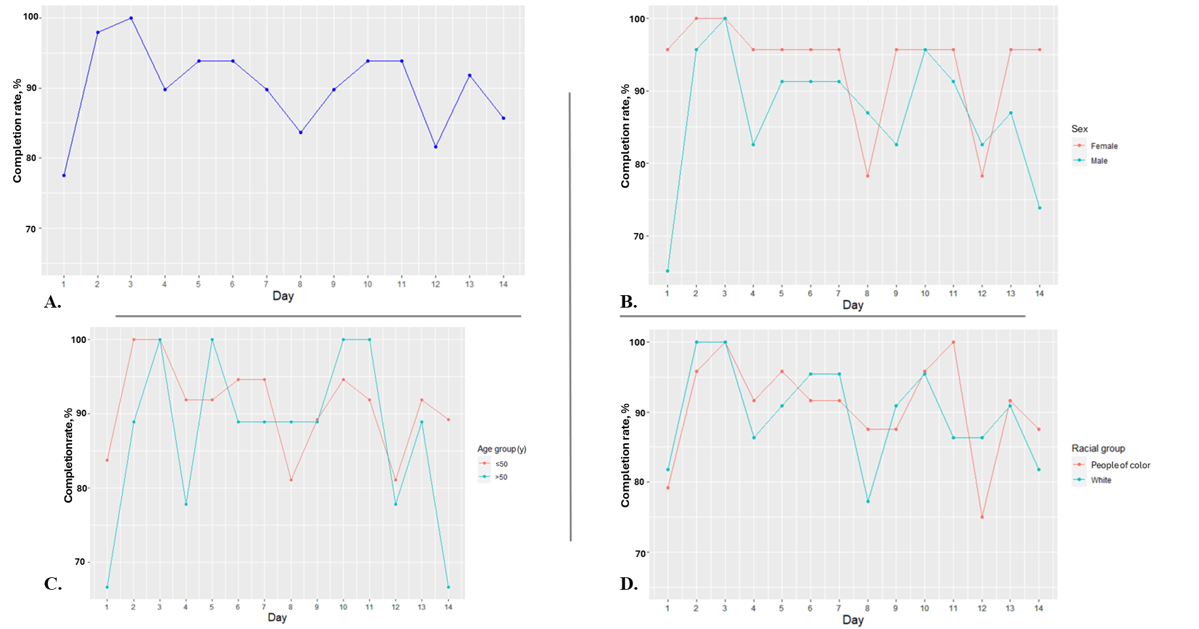
Self-report completion rates of participants (A) over time (n=49) and stratified by (B) sex (n=46), (C) age group (n=46), and (D) racial group (n=46).

Concerning fidelity, Figure 3A shows the self-report completion rates of participants (n=49) over time, which ranged from 78% (38/49; day 1) to 100% (49/49 day 3) during the 14-day follow-up. The target threshold of 75% was met at each time point. The completion rates exhibited a slight tendency to decrease over time, but these results were not statistically significant (*P*=.21).

Figure 3B shows the completion rates over time, stratified by sex (n=46). For female participants (23/46, 50%), they ranged from 78% (18/23; day 8) to 100% (23/23; days 2 and 3). For male participants (23/46, 50%), they ranged from 65% (15/23; day 1) to 100% (23/23; day 3). Overall, female participants had a significantly higher fidelity rate over time than male participants (*P*=.004).

Completion rates by age group over time (n=46) are shown in Figure 3C. They ranged from 81% (30/37; days 8 and 12) to 100% (37/37; days 2 and 3) for participants aged 50 years (37/46, 80%). Among participants aged >50 years (9/46, 20%), completion rates ranged from 67% (6/9; days 1 and 14) to 100% (9/9; days 3, 5, 10, and 11). No significant difference was found in fidelity between the 2 age groups over time (*P*=.19).

Figure 3D shows completion rates over time stratified by racial group (n=46). For participants of color (24/46, 52%), they ranged from 75% (18/24; day 12) to 100% (24/24; days 3 and 11). For White participants (22/46, 48%), they ranged from 77% (17/22; day 8) to 100% (22/22; days 2 and 3). There was no significant difference in fidelity between the 2 racial groups over time (*P*=.72).

Table 2 displays the descriptive statistics for acceptability and usability at each time point for the overall sample, as well as for the sample stratified by sex, age, and racial group. For the overall sample, the *P* values correspond to the null hypothesis of no mean difference between time points (day 1, day 7, and day 14 of follow-up). For the sociodemographic group comparisons, the *P* values correspond to the null hypothesis of no mean difference between groups.

**Table 2:** Descriptive statistics for the intervention’s acceptability and usability scores at each time point for the overall sample (n=49) and stratified by the sociodemographic variables (n=46).

Sociodemographic variables				Total (n=49)		Sex				Age group (years)				Racial group		
						Female (n=23)		Male (n=23)		18-50 (n=37)		51-70 (n=9)		People of color (n=24)		White (n=22)
**Acceptability (Acceptability of Intervention Measure)**																
	Day 1, mean (SD; min-max)			4.06 (0.57; 1.75-5.00)		4.00 (0.65; 1.75-5.00)		4.14 (0.49; 3.00-5.00)		4.02 (0.57; 1.75-5.00)		4.22 (0.58; 3.25-5.00)		4.08 (0.70; 1.75-5.00)		4.05 (0.41; 3.00-5.00)
	Day 7, mean (SD; min-max)			4.26 (0.59; 2.75-5.00)		4.32 (0.61; 2.75-5.00)		4.25 (0.49; 3.25-5.00)		4.29 (0.55; 2.75-5.00)		4.28 (0.60; 3.25-5.00)		4.33 (0.59; 2.75-5.00)		4.24 (0.52; 3.25-5.00)
	Day 14, mean (SD; min-max)			4.23 (0.57; 2.75-5.00)		4.32 (0.71; 2.75-5.00)		4.21 (0.43; 3.25-5.00)		4.28 (0.59; 2.75-5.00)		4.19 (0.61; 3.25-5.00)		4.26 (0.60; 2.75-5.00)		4.26 (0.57; 3.00-5.00)
	*P* value			.04^a^		.99^b^		.99^b^		.90^b^		.90^b^		.88^b^		.88^b^
**Usability**																
	**Perceived impact**															
		Day 1, mean (SD; min-max)	4.34 (0.71; 2.67-5.00)		4.25 (0.73; 2.67-5.00)		4.41 (0.71; 3.00-5.00)		4.32 (0.64; 2.67-5.00)		4.33 (1.00; 3.00-5.00)		4.44 (0.68; 3.00-5.00)		4.21 (0.75; 2.67-5.00)	
		Day 7, mean (SD; min-max)	4.49 (0.66; 2.67-5.00)		4.48 (0.74; 2.67-5.00)		4.46 (0.60; 3.33-5.00)		4.45 (0.66; 2.67-5.00)		4.54 (0.75; 3.33-5.00)		4.56 (0.65; 2.67-5.00)		4.38 (0.69; 2.67-5.00)	
		Day 14, mean (SD; min-max)	4.40 (0.82; 1.00-5.00)		4.44 (0.90; 1.00-5.00)		4.30 (0.78; 2.33-5.00)		4.50 (0.76; 1.00-5.00)		3.85 (0.96; 2.33-5.00)		4.25 (0.92; 1.00-5.00)		4.50 (0.74; 2.33-5.00)	
		*P* value	.80^a^		.72^b^		.72^b^		.27^b^		.27^b^		.68^b^		.68^b^	
	**Usefulness**															
		Day 1, mean (SD; min-max)	4.66 (0.47; 3.00-5.00)		4.66 (0.45; 3.67-5.00)		4.65 (0.51; 3.00-5.00)		4.69 (0.40; 3.67-5.00)		4.53 (0.72; 3.00-5.00)		4.63 (0.56; 3.00-5.00)		4.68 (0.38; 3.67-5.00)	
		Day 7, mean (SD; min-max)	4.68 (0.39; 3.56-5.00)		4.70 (0.40; 3.56-5.00)		4.66 (0.41; 3.78-5.00)		4.71 (0.36; 3.56-5.00)		4.53 (0.54; 3.78-5.00)		4.68 (0.42; 3.78-5.00)		4.68 (0.39; 3.56-5.00)	
		Day 14, mean (SD; min-max)	4.60 (0.68; 1.00-5.00)		4.57 (0.90; 1.00-5.00)		4.62 (0.46; 3.67-5.00)		4.65 (0.72; 1.00-5.00)		4.37 (0.61; 3.67-5.00)		4.47 (0.90; 1.00-5.00)		4.73 (0.36; 3.89-5.00)	
		*P* value	.50^a^		.76^b^		.76^b^		.13^b^		.13^b^		.82^b^		.82^b^	
	**Ease of use**															
		Day 1, mean (SD; min-max)	4.60 (0.57; 3.00-5.00)		4.61 (0.51; 3.60-5.00)		4.58 (0.65; 3.00-5.00)		4.67 (0.45; 3.60-5.00)		4.29 (0.91; 3.00-5.00)		4.49 (0.64; 3.00-5.00)		4.71 (0.49; 3.20-5.00)	
		Day 7, mean (SD; min-max)	4.67 (0.53; 2.60-5.00)		4.76 (0.37; 4.00-5.00)		4.53 (0.67; 2.60-5.00)		4.77 (0.35; 4.00-5.00)		4.13 (0.89; 2.60-5.00)		4.59 (0.61; 2.60-5.00)		4.71 (0.47; 3.20-5.00)	
		Day 14, mean (SD; min-max)	4.64 (0.71; 1.00-5.00)		4.64 (0.88; 1.00-5.00)		4.61 (0.56; 3.20-5.00)		4.72 (0.71; 1.00-5.00)		4.24 (0.71; 3.20-5.00)		4.52 (0.94; 1.00-5.00)		4.73 (0.41; 3.80-5.00)	
		*P* value	.79^a^		.69^b^		.69^b^		.004^b^		.004^b^		.30^b^		.30^b^	

^a^*P* value corresponds to the null hypothesis of no mean difference between time points (days 1, 7, and 14 of follow-up).

^b^*P* value corresponds to the null hypothesis of no mean difference between groups.

The mean values of acceptability and usability were above the set minimum of 4 at each time point. Their means increased between day 1 and day 7 and stabilized or slightly decreased between day 7 and day 14. Mean acceptability scores differed significantly over time (*P*=.04); they increased from day 1 to day 7 (*P*=.04) and from day 1 to day 14 (*P*=.07). No significant differences were found in mean usability scores between time points.

Mean acceptability scores surpassed the required minimum of 4 at each time point for all sociodemographic groups. No significant differences were found in mean acceptability scores between groups, for each variable considered, independent of time point.

Mean usability scores were above the minimum threshold of 4 at each time point and for each sociodemographic variable. No significant differences in mean usability scores were found between groups, independent of time point, except for ease of use. In this case, participants aged >50 years reported significantly lower mean ease of use scores than younger participants (*P*=.004).

In addition, among the 49 patients who completed the 14-day follow-up, 3 (6%) were not able to complete the daily self-report by themselves and needed help from someone to complete it for at least 1 day.

#### Perceived Response Burden

Table 3 displays the descriptive statistics for the perceived response burden of completing the daily self-reports at each time point for the overall sample and stratified by sex, age, and racial group. For the overall sample, the *P* value corresponds to the null hypothesis of no effect of time on the odds of being at a lower burden level, while for the sociodemographic group comparisons, the *P* values refer to the null hypothesis of no difference between groups in the odds of being at a lower response burden level.

**Table 3:** Descriptive statistics for intervention-related response burden at each time point for the overall sample (n=49) and stratified by the sociodemographic variables (n=46).

Day and response ^a^		Total (n=49), n (%)	Sex		Age group (years)		Racial group	
			Female (n=23), n (%)	Male (n=23), n (%)	18-50 (n=37), n (%)	51-70 (n=9), n (%)	People of color (n=24), n (%)	White (n=22); n (%)
**Day 1**								
	1	22 (45)	10 (43)	11 (48)	17 (46)	4 (44)	10 (42)	11 (50)
	2	20 (41)	11 (48)	9 (39)	17 (46)	3 (33)	11 (46)	9 (41)
	3	3 (6)	2 (9)	1 (4)	2 (5)	1 (11)	1 (4)	2 (9)
	4	1 (2)	0 (0)	1 (4)	0 (0)	1 (11)	1 (4)	0 (0)
	5	0 (0)	0 (0)	0 (0)	0 (0)	0 (0)	0 (0)	0 (0)
	Missing	3 (6)	0 (0)	1 (4)	1 (3)	0 (0)	1 (4)	0 (0)
**Day 7**								
	1	31 (63)	16 (70)	13 (57)	25 (68)	4 (44)	11 (46)	18 (82)
	2	16 (33)	7 (30)	8 (35)	11 (30)	4 (44)	11 (46)	4 (18)
	3	0 (0)	0 (0)	0 (0)	0 (0)	0 (0)	0 (0)	0 (0)
	4	0 (0)	0 (0)	0 (0)	0 (0)	0 (0)	0 (0)	0 (0)
	5	0 (0)	0 (0)	0 (0)	0 (0)	0 (0)	0 (0)	0 (0)
	Missing	2 (4)	0 (0)	2 (9)	1 (3)	1 (11)	2 (8)	0 (0)
**Day 14**								
	1	32 (65)	17 (74)	12 (52)	24 (65)	5 (56)	15 (63)	14 (64)
	2	13 (27)	4 (17)	9 (39)	11 (30)	2 (22)	7 (29)	6 (27)
	3	3 (6)	1 (4)	2 (9)	1 (3)	2 (22)	1 (4)	2 (9)
	4	0 (0)	0 (0)	0 (0)	0 (0)	0 (0)	0 (0)	0 (0)
	5	0 (0)	0 (0)	0 (0)	0 (0)	0 (0)	0 (0)	0 (0)
	Missing	1 (2)	1 (4)	0 (0)	1 (3)	0 (0)	1 (4)	0 (0)
*P* value^c^		.21^b^	.67^c^	.67^c^	.49^c^	.49^c^	.23^c^	.23^c^

^a^1=“very easy,” 2=“quite easy,” 3=“neither easy nor burdensome,” 4=“quite burdensome,” and 5=“very burdensome.” “Missing” corresponds to the missing value at each time point.

^b^*P* value corresponds to the null hypothesis of no effect of time on the odds of being at a lower burden level.

^c^*P* values refer to the null hypothesis of no difference between groups in the odds of being at a lower response burden level.

Surpassing our target, >80% of the participants at each time point—86% (42/49) at day 1, 96% (47/49) at day 7, and 92% (45/49) at day 14—rated completing the daily self-reports (response burden) as “quite easy” to “very easy,” with no significant differences found between time points (*P*=.21).

Approximately 80% of the participants in each sociodemographic group found it “very easy” or “quite easy” to complete the daily self-reports, with no significant difference between groups, independent of time point.

### Qualitative Results

A total of 13 individuals participated in the interviews from June to September 2021, including engaged expert patients (n=3, 23% women who had recovered from COVID-19 infection), health care professionals (n=6, 46%, including the PI of the study; n=3, 50% research nurses; and n=2, 33% physicians), and the coordinators (n=4, 31%, including n=1, 25% clinical study coordinator; n=1, 25% digital coordinator; n=1, 25% laboratory coordinator; and n=1, 25% Opal manager). The thematic analysis identified 8 themes of factors that influenced the implementation process related to 10 constructs across the 5 domains of the CFIR.

#### Implementation Process

##### Planning: Structured Process

For the interviewed stakeholders, meetings held during the “solution design” phase (Figure 1) enhanced feasibility by providing guidance and structure in the intervention’s preparation, mainly by turning this process into a series of manageable collective decisions about its key components, as mentioned by a participant:

Meetings were quite structured. We had Zoom meetings to discuss the questionnaire, and the questions to include, details that we had to review...I think these steps were well placed and framed, so the project could be launched and useful for people.

Expert patient 1

##### Planning: Focus on Stakeholder Recommendations

Stakeholders highlighted the presence of different experts at these meetings, including expert patients, physicians, nurses, a psychiatrist, and IT developers, and how the integration of their perspectives improved quality and patient centeredness. The same expert patient stated as follows:

We were several people revising documents. We made other revisions. Then we applied it and tested it physically. And we gave feedback during Zoom meetings with the two nurses, and I was there as a “patient.” We could look at it, and answer questions, and then [IT developers] would do their thing making sure that it is “user-friendly.”

Expert patient 1

##### Engaging: Emphasis on Stakeholder Knowledge

Stakeholders described their involvement as a learning process and an opportunity to share experience and expertise related to COVID-19 infection, medical follow-up, and IT. The implementation process led to a series of mutual training that benefited stakeholders and facilitated feasibility:

Since I am part of the team, they [health care professionals] provided me with the overview, the importance, and the significance of the study, which is very interesting.

Coordinator; laboratory coordinator

The principal investigator is a physician with whom we work, in the same clinic. We are three research nurses, linked to an outpatient clinic. We see patients in other research projects from the clinic, so we know about this kind of research and could share our experience. As other stakeholders, he approached us, and it made a motivated team for the implementation. It included the patient committee, which helped a lot, because they could test the app.

Health care professional; nurse 3

#### Intervention

##### Security: Burdensome Privacy Protection Measures

Stakeholders who interacted with patient participants in the pilot study mentioned that many had felt that these measures were cumbersome or time consuming; for instance, a stakeholder stated as follows:

What patients disliked the most were the security measures. They really disliked having to use such a complex password with low and upper case, with special characters.

Coordinator; clinical research coordinator

This aspect was identified as a barrier to usability, especially ease of use.

##### Relative Advantage: Emphasis on Providing Safe Care for All

Several stakeholders commented on how patient safety was set as a priority throughout the implementation process, and this aspect was identified as enhancing acceptability. They highlighted the importance of both patients and health care professionals feeling secure with the technology and the intervention throughout the follow-up. Indeed, a stakeholder presented the whole project as having emerged from a concern for the safety of self-isolating patients with COVID-19 infection:

[We had an initial meeting] to discuss basically the algorithm and what steps should be taken to provide safe and good care in the event that a patient decompensates, who should be notified, should they just be informed to go to the emergency.

Health care professional; physician 1

In this vein, a stakeholder, a patient expert who was also a health care professional, discussed the intervention as reassuring from both the patient and professional perspectives:

For me, as a nurse, I found it reassuring to touch base every day with these patients, because they could deteriorate very, very quickly. Often, we wait for them to call, or for their next appointment, it can be a long time and there can be changes. These questionnaires ask relevant questions on the condition itself. The nurse can see it: “They’re deteriorating. I want to talk with them. I want to understand what is going on...” As a patient, I find reassuring that my health care team knows what is happening to me, and I do not have to wait to become very sick to go to the emergency room. And if I’m worried, I can leave a message.

Expert patient 2

##### Relative Advantage: Reduced Stress

Stakeholders commented that the intervention was instrumental in providing patient participants with emotional support and reducing stress associated with their health status. This aspect, identified as facilitating usefulness, was attributed to the access acquired via the intervention to health care professionals and to reliable information on COVID-19:

I think that [the information on COVID-19 within Opal] is a big addition for patients because they are at home, with COVID-19. They have many worries, with everything they hear in the media. So, it could reassure them. And to have somebody on the phone to answer questions, I think it’s really something good that could calm them and ease their preoccupations.

Health care professional; nurse 3

##### Adaptability: Certain Functionalities Require Further Tailoring for Acute Follow-Up

Stakeholders explained that Opal was not perfectly adapted to closely monitor acute conditions such as COVID-19 infection (eg, administration and collection of daily surveys) probably because it had been conceived for the clinical follow-up of chronic conditions (eg, no integrated automated reminder system). They consequently took measures to encourage patient participants to adhere to the intervention to optimize fidelity. Mainly, study coordinators and health care professionals often sent in-app text message reminders to patient participants for them to complete daily self-reports, resulting in increased workloads:

Before this project, Opal was used mainly by cancer patients...As I said, even if the system allows to distribute surveys, it’s not very easy because it’s not conceived to use questionnaires to monitor patients, at least [not] every day. It’s better for chronic conditions with one consultation once every second or third week.

Coordinator; digital coordinator

##### Adaptability: Adjustable Intervention to Meet Emerging Needs

Stakeholders appreciated that the intervention could be adjusted to individual patient needs, which enhanced usability; for instance, the duration of participation was extended for certain participants based on perceived risks (eg, when they were infected with an emerging COVID-19 variant); some participants were invited to answer >1 self-report on certain days to better monitor their state (eg, if they showed risks of rapid deterioration); 2 (4%) of the 49 participants applied questions about symptoms, vital signs, and mental health to other household members (eg, their children) with COVID-19 infection—without transmitting them to health care professionals—who could not participate in the study to enable monitoring; and patient participants and health care professionals used telephone consultations more often than expected and for unanticipated purposes (eg, to discuss a chronic health condition, learn how to use the oximeter, and discuss remote work conditions in the context of self-isolation):

Flexibility, this is what people enjoyed the most. Most of all when patients were sicker. Even if the protocol planned for a 14-day follow-up, we extended this follow-up with three patients. One woman was very worried about her son, but the son was excluded because he was not an adult. But being in Opal, she felt reassured as she could use the questions used in Opal, and also employed the oximeter on her son.

Coordinator; clinical research coordinator

Patient participants reappropriated the study. We hadn’t planned that they would call the nurses all the time. It was not planned at all. And there were contacts outside of the application. This is what is interesting, they wanted to talk to the nurse, and have Zoom consultations.

Health care professional; PI

#### Inner and Outer Settings

##### Networks and Communication: Delays Due to Institutional Barriers

For stakeholders, an important barrier to feasibility was the institutional approval process for Opal’s protection measures to ensure patient privacy and data security. A coordinator noted as follows:

We were delayed by the ethics committee because of concerns about the safety of patient data. The committees were very worried, and they put a lot of conditions.

Coordinator; digital coordinator

Another stakeholder provided the following comment:

We were completely blocked by the security department, an internal MUHC institution responsible for validating all “IT tools.” I think they blocked us for about four months. We could not submit the project to Ethics, we could not finish it. We only received ethics approval in December, and the approval from this institution within the MUHC, and this is why we started in December to include patients. Otherwise, we could have started before.

Health care professional; PI

These measures delayed the institutional approval for the implementation of the intervention, as well as the recruitment of patient participants.

## Discussion

### Principal Findings

This paper reports on the implementation of a patient portal (Opal) configured to support the follow-up of self-isolating patients with COVID-19 infection. A mixed methods pilot study was conducted to test and evaluate the intervention’s implementation with 49 patient participants who used Opal for at least 14 days. Quantitatively, the implementation was evaluated with research questionnaires administered to patients on the intervention’s acceptability, usability (including perceived impact, usefulness, and ease of use), and perceived response burden, as well as through descriptive statistics on feasibility and fidelity. Qualitatively, semistructured interviews on implementation barriers and facilitators were held with 13 stakeholders of the intervention, including expert patients, health care professionals, and coordinators.

The COVID-19 pandemic has led to the development of numerous remote monitoring programs to support patients as well as health care systems. Initially focused on discharge follow-up of admitted patients [48-50], the technology was expanded to direct remote monitoring of patients in home isolation who had tested positive for COVID-19 infection and those who were suspected of having been infected with COVID-19 [51-58]. Multiple studies have shown that such interventions can help patients better manage their symptoms at home and reduce patient hospitalization or rehospitalization rates [48-50,53,54,56,57]. Patients could also be identified and admitted in a timelier manner after their condition worsened, reflecting the fact that remote monitoring programs are a good way to ease the management of hospital beds and reduce the burden on the health care system during a pandemic [52,55-58]. However, 2 systematic reviews on COVID-19 remote home monitoring programs noted the lack of implementation research on these technologies and attention to stakeholder perspectives [59,60]. Our study fills this gap by describing PSE throughout the implementation process and by analyzing stakeholder experiences, providing evidence for co-design through PSE.

Health information technologies have the potential to increase access to health care, but digital divides related to limited access to technology or technological literacy may alienate certain groups, such as women, older people, or certain ethnic or racialized groups [61,62]. The literature highlights the need to consider equity when implementing telehealth interventions and reduce these divides [63,64]. In this regard, the sociodemographic profiles of the pilot study participants were relatively diverse: nearly half (23/49, 47%) were female, close to half (24/49, 49%) were people of color, and almost a fifth (9/49, 18%) were aged >50 years. Nevertheless, we achieved the minimum success thresholds set for all included implementation outcomes (ie, fidelity, feasibility, acceptability, usability, and perceived response burden), at all time points and across all sociodemographic groups considered. Hence, we can conclude that the intervention was feasible in the context of implementation.

The positive feasibility and fidelity results support the intervention’s viability. The 98% (51/52) recruitment rate indicates that self-isolating patients wanted to stay connected to the health system, which the Opal patient portal’s smartphone app allowed. Given the delays caused by institutional barriers in both solution deployment and patient recruitment, we started the study only during the second wave of COVID-19 in Quebec, and participants were often enrolled on or after the third day of a positive test confirmation. The team was concerned that the 14-day routine follow-up, initially chosen according to official guidelines, was too long. Research suggests that half of those who download mobile health apps stop using them because of loss of interest, high data entry burden, or hidden costs [65]. While we observed a slight decrease in fidelity over time, this change was not significant. It was also found that female participants had significantly higher fidelity over time than male participants (*P*=.004). This is consistent with research that suggests that women are usually more concerned about health issues and more likely to report their health care problems than men [66]. In sum, the 96% (49/51) retention rate and >80% response rate to the self-reports exceeded researchers’ expectations. The Opal intervention for COVID-19 seems to have responded well to the needs of the target population, and patient and stakeholder involvement throughout the configuration and implementation process likely contributed to the positive feasibility and fidelity results.

One explanation for the high retention rate may lie in the participants’ high acceptability ratings of the intervention, which, in turn, may have been fostered by the co-design approach taken in the planning phase. This allowed the intervention to be refined based on suggestions from a range of perspectives and areas of expertise. Previous studies have underscored the ability of PSE to improve the acceptability of studies [60,67], and our results suggest its utility for telehealth intervention–based studies. Furthermore, in situations where participants are self-isolating and have little knowledge of COVID-19, it is important to design interventions with their safety as a primary concern. In this case, the intervention contributed to ensure safety, which also contributed to its acceptability. Similarly, it is worth noting the significant increase in the acceptability score observed between day 1 and day 7 (from mean 4.06, SD 0.57 to mean 4.26, SD 0.59). This increase resonates with models of acceptance of health IT that imply that the use of the technology contributes to acceptance [68]. In other words, acceptability tends to increase over time as users learn to use a technology and if they are satisfied by its quality and the services it provides [69].

The usability of Opal for COVID-19 remote follow-up was also demonstrated. Our results indicate that it was impactful, useful, and easy to use. We only noted a significant difference on this outcome for 1 sociodemographic variable: age. People aged >50 years showed significantly lower mean ratings of ease of use than younger participants. While the sample size of this age group was very small (9/46, 20%), the qualitative results suggest that the rigorous privacy protection mechanisms were an implementation barrier. Opal has a complex password combination requirement, which can be challenging for older users. Moreover, Opal automatically logs users out of their accounts if they are inactive for >5 minutes. This can result in users having to log in repeatedly to complete self-reports if they are interrupted. The balance between usability and security could be further considered in the future [70].

By contrast, our qualitative results, particularly the themes identified for the “adaptability” construct, suggest that usability was contingent on the reactiveness of stakeholders; for example, health care professionals offered more teleconsultations than expected and, in some cases, provided support to patients’ family members who also had contracted COVID-19 infection. Furthermore, the technical team sent more reminders than expected to patients to fill out their daily self-reports. Indeed, timely feedback and support to users is important to ensure the usability of telehealth technologies [71]. Similar to a previous study [72], this may increase stakeholder workload, especially when there is still room for improvement in the technology. However, our qualitative results suggest that these adaptations were not seen as a burden to stakeholders. The literature on the impacts of such tools on workload suggests that any extra effort by service providers may be compensated by an increased ability to identify information that would otherwise have been missed and intervene early to avoid worse outcomes [73,74]. What may have occurred instead was a reprioritization of work time [73,74]. Such benefits were also seen by stakeholders as part of the ultimate purpose of this project, which was to increase access to care, promote safety, and reduce mental stress for homebound, self-isolating patients with COVID-19 infection. Nonetheless, on the technical front, future implementation will require more advanced automation of such features as reminders. Further assessment of the costs associated with potential large-scale implementation of this intervention, including workforce requirements, is also recommended. Future studies could analyze its cost-effectiveness and, to facilitate more efficient staffing, document the reorientation of human interactions necessitated when using a patient portal.

It is finally worth noting that >80% of the patient participants found it “quite easy” or “very easy” to answer the self-reports via Opal. Overall, 94% (46/49) of the patient participants were able to complete daily self-reports by themselves. Both results illustrate the feasibility of sharing information with the health care team through the patient portal by answering electronically administrated PROMs in mild COVID-19 infection conditions, further supporting the usability of the intervention.

### Limitations

We acknowledge several limitations of this study. First, the sample size of this pilot study was small. Furthermore, participants were recruited through convenience sampling, potentially contributing to sampling bias; for instance, participants may have been more willing to participate and to rate the intervention favorably. Patient participants were also screened at a single institution in Montreal. Therefore, the generalizability of our findings to other geographic areas is limited. Future similar studies should consider increasing their sample size, adding a control group (eg, a control group that only receives daily telehealth check-ins from health care providers), and including multiple study sites to enhance the reliability of their findings.

Second, for technical reasons related to participant identification, enrollment in Quebec’s provincial health insurance plan was necessary for inclusion in the study, which led us to exclude 4 potential patient participants who were members of populations considered vulnerable during the COVID-19 pandemic (eg, international students and resettled refugees) [75,76]. As such, the intervention may have contributed to health care inequity. Future improvements to the identification system could help alleviate this issue.

Finally, the qualitative interviews with stakeholders were conducted 1 month after completing quantitative data collection, which may have introduced recall bias.

### Conclusions

This work illustrates how PSE can enable co-design, including the development and implementation of a telehealth intervention for remote follow-up of an emerging acute condition (ie, COVID-19 infection), in this case, by making configurational changes to a patient portal used for chronic disease management. The mixed methods pilot study design provided a detailed understanding of the positive implementation outcomes of the intervention and identified some barriers. Thresholds were attained or surpassed for the feasibility, fidelity, usability, acceptability, and perceived response burden of the intervention, and the qualitative findings highlighted the importance of PSE in the configuration and implementation processes. These data also further demonstrate the significant potential of such telehealth tools for managing acute but stable illnesses or medical conditions that require remote follow-up. Future work can be devoted to further tailoring such interventions, improving the balance of usability and security measures, and assessing the cost of large-scale implementation.

## Appendix

Multimedia Appendix 1 Opal-COVID study daily self-report.

Multimedia Appendix 2 CONSORT (Consolidated Standards of Reporting Trials) feasibility guidelines.

Multimedia Appendix 3 StaRI (Standards for Reporting Implementation Studies) guidelines.

Multimedia Appendix 4 Study questionnaire (adapted Acceptability of Intervention Measure, Health Information Technology Usability Evaluation Scale, and questionnaire for perceived response burden).

Multimedia Appendix 5 Qualitative interview guide.

Multimedia Appendix 6 Sociodemographic characteristics of the study sample.

## Abbreviations

CFIR: Consolidated Framework for Implementation Research
CONSORT: Consolidated Standards of Reporting Trials
MUHC: McGill University Health Centre
PI: principal investigator
PROM: patient-reported outcome measure
PSE: patient and stakeholder engagement
StaRI: Standards for Reporting Implementation Studies

## Data Availability

The datasets generated and analyzed during this study are available from the corresponding author on reasonable request.
