# Supplementary material for: Implementing a Patient Portal for the Remote Follow-Up of Self-Isolating Patients With COVID-19 Infection Through Patient and Stakeholder Engagement (the Opal-COVID Study): Mixed Methods Pilot Study
Source: J Particip Med. 2024 Dec 4;16:e48194. doi: 10.2196/48194 (PMC11656113; doi:10.2196/48194)
Supplement: Multimedia Appendix 4 [file jopm_v16i1e48194_app4.doc]

**Study questionnaire on the feasibility of the Opal app**

**Your opinion on the Opal smartphone app**

Indicate your agreement or disagreement with the following statements about the smartphone app. When you think about the app, think also about what you are required to do with the app and the services it provides.

|  | Completely disagree | Disagree | Neither agree nor disagree | Agree | Completely agree |
| --- | --- | --- | --- | --- | --- |
| 1. The app meets my approval. | ➀ | ➁ | ➂ | ➃ | ➄ |
| 2. The app is appealing to me. | ➀ | ➁ | ➂ | ➃ | ➄ |
| 3. I like the app. | ➀ | ➁ | ➂ | ➃ | ➄ |
| 4. I would recommend the app. | ➀ | ➁ | ➂ | ➃ | ➄ |

|  | Strongly disagree |  |  |  | Strongly agree |
| --- | --- | --- | --- | --- | --- |
| I think the app is a positive addition for people at home with COVID-19 | ➀ | ➁ | ➂ | ➃ | ➄ |
| I think the app improves my quality of life, while at home with COVID-19 | ➀ | ➁ | ➂ | ➃ | ➄ |
| The app is an important part of looking after me, while I am at home with COVID-19 | ➀ | ➁ | ➂ | ➃ | ➄ |
| Using the app makes it easier to stay on top of my COVID-19 | ➀ | ➁ | ➂ | ➃ | ➄ |
| Using the app enables me to inform healthcare providers about my health more quickly | ➀ | ➁ | ➂ | ➃ | ➄ |
| Using the app makes it more likely that I will be checked to see if my health declines | ➀ | ➁ | ➂ | ➃ | ➄ |
| Using the app is useful for connecting me with a healthcare team | ➀ | ➁ | ➂ | ➃ | ➄ |
| I think the app is an accessible tool for people at home with COVID-19 | ➀ | ➁ | ➂ | ➃ | ➄ |
| I am satisfied with the app for keeping track of people at home with COVID-19 | ➀ | ➁ | ➂ | ➃ | ➄ |
| I provide health information to the healthcare team in a timely manner because of the app | ➀ | ➁ | ➂ | ➃ | ➄ |
| Using the app increases the supervision of my COVID-19 | ➀ | ➁ | ➂ | ➃ | ➄ |
| I am able to find useful information about COVID-19 with the app | ➀ | ➁ | ➂ | ➃ | ➄ |
| I am confident in my ability to use the app | ➀ | ➁ | ➂ | ➃ | ➄ |
| Learning to operate the app is easy for me | ➀ | ➁ | ➂ | ➃ | ➄ |
| It is easy for me to become skillful at using the app | ➀ | ➁ | ➂ | ➃ | ➄ |
| I find the app easy to use | ➀ | ➁ | ➂ | ➃ | ➄ |
| I can remember how to use the app | ➀ | ➁ | ➂ | ➃ | ➄ |

|  | Very easy | Quite easy | Neither easy nor burdensome | Quite burdensome | Very burdensome |
| --- | --- | --- | --- | --- | --- |
| How was it for you to complete the daily questionnaire for the healthcare team? | ➀ | ➁ | ➂ | ➃ | ➄ |
