# Supplementary material for: Implementing a Patient Portal for the Remote Follow-Up of Self-Isolating Patients With COVID-19 Infection Through Patient and Stakeholder Engagement (the Opal-COVID Study): Mixed Methods Pilot Study
Source: J Particip Med. 2024 Dec 4;16:e48194. doi: 10.2196/48194 (PMC11656113; doi:10.2196/48194)
Supplement: Multimedia Appendix 6 [file jopm_v16i1e48194_app6.pdf]

Table 1 Sociodemographic characteristics of the study sample

		n (%)
Sex	Female	23 (47%)
	Male	23 (47%)
	Missing	3 (6%)
Age group	18-30	13 (27%)
	31-40	13 (27%)
	41-50	11 (22%)
	51-60	6 (12%)
	61-70	3 (6%)
	71-80	0 (0%)
	Over 80	0 (0%)
	Missing	3 (6%)
Current occupation	Paid employment	28 (57%)
	Unemployed	8 (16%)
	Student	3 (6%)
	Sick leave or work-related illness/injury leave due to COVID-19	3 (6%)
	Homemaker, Retired, Sick leave or work-related illness/injury leave not due to COVID-19, or Long-term disability	0 (0%)
	Other	4 (8%)
	Missing	3 (6%)
Racial group	White	22 (45%)
	Black	6 (12%)
	Indian/South-Asian	5 (11%)
	Aboriginal/First Nations	0 (0%)
	North African/Middle Eastern	1 (2%)
	Asian/Pacific Islander	3 (6%)
	Latin American	3 (6%)
	Other	6 (12%)
	Missing	3 (6%)
